# Prediction of Gastric Cancer-Related Genes Based on the Graph Transformer Network

**DOI:** 10.3389/fonc.2022.902616

**Published:** 2022-06-30

**Authors:** Yan Chen, Xuan Sun, Jiaxing Yang

**Affiliations:** Department of Gastrointestinal Surgery, The First Hospital of Jilin University, Changchun, China

**Keywords:** gastric cancer, susceptibility gene, graph transformer network, deep belief network, heterogeneous network

## Abstract

Gastric cancer is a complex multifactorial and multistage process that involves a large number of tumor-related gene structural changes and abnormal expression. Therefore, knowing the related genes of gastric cancer can further understand the pathogenesis of gastric cancer and provide guidance for the development of targeted drugs. Traditional methods to discover gastric cancer-related genes based on biological experiments are time-consuming and expensive. In recent years, a large number of computational methods have been developed to identify gastric cancer-related genes. In addition, a large number of experiments show that establishing a biological network to identify disease-related genes has higher accuracy than ordinary methods. However, most of the current computing methods focus on the processing of homogeneous networks, and do not have the ability to encode heterogeneous networks. In this paper, we built a heterogeneous network using a disease similarity network and a gene interaction network. We implemented the graph transformer network (GTN) to encode this heterogeneous network. Meanwhile, the deep belief network (DBN) was applied to reduce the dimension of features. We call this method “DBN-GTN”, and it performed best among four traditional methods and five similar methods.

## Introduction

Gastric cancer is a malignant tumor originated from gastric mucosal epithelial cells (1). At present, due to the increase of work pressure, the change of diet structure, and *Helicobacter pylori* infection, gastric cancer is gradually showing a younger trend (2, 3). Patients with early gastric cancer often have no obvious symptoms, or only nonspecific symptoms such as abdominal discomfort and flatulence (4). These symptoms are often similar to chronic gastric symptoms such as dyspepsia, gastritis, and gastric ulcer (5). Most patients with early-stage cancer find their condition through gastroscopy. Under reasonable medical measures, the 5-year survival rate of patients with early-stage gastric cancer can reach 90% (6). However, most patients with gastric cancer are in the middle and late stage of gastric cancer when they are diagnosed. The tumor has invaded the outside of the stomach and is complicated by lymph node metastasis; thus, the odds of being cured is low. Screening related genes closely related to gastric cancer can be used as molecular targets for diagnosis (7). Different gene combinations can reflect the early diagnosis, incidence, effectiveness of treatment, and prognosis of gastric cancer. The early stage of gastric cancer generally only contains a few gene changes. These changes are potential molecular targets for early diagnosis (8). If these changes can be detected, gastric cancer can be detected as soon as possible, which can greatly improve the cure rate of gastric cancer. The typing of gastric cancer susceptibility genes and related genes can also provide some information for the prediction of the disease (9), so as to take preventive measures as soon as possible to prevent the deterioration of the disease. With the continuous in-depth post-genome studies, more genotypes will be found to be related to the occurrence, development, and prognosis of gastric cancer. The final conclusion can provide a new theoretical basis for the discussion of the molecular mechanism of gastric cancer (10, 11).

Gastric cancer is a complex and multifactorial disease. Environmental and genetic factors play an important role in the occurrence of gastric cancer (12, 13). MiRNA precisely regulates the occurrence of gastric cancer by participating in a network system composed of a series of important biological processes such as cell proliferation, apoptosis, and differentiation (14). A large number of studies have shown that according to the difference in expression level, specific miRNAs have become a potential biomarker of malignant cancer and have an impact similar to carcinogenic or tumor suppressor genes. For example, the expression of miR-21 and miR-155 is usually increased in gastric cancer, which can promote cell proliferation and induce the occurrence of malignant cancer (15), and the expression of mir-449 is usually reduced. It can inhibit cell proliferation and inhibit the further development of gastric cancer (16). To a large extent, miRNA is almost involved in the whole process of gastric cancer pathogenesis. Therefore, with the deepening of research, it can enrich the biological function of miRNA, show a new vision for the in-depth study of the molecular mechanism of the occurrence and development of gastric cancer, and show a broader platform for the medical field. The application of gene chips can further extend the research on gastric cancer into the gene regulation network, making it possible to explore the gene expression profile of gastric cancer in different pathological stages. Gene chips have become a powerful tool to study the molecular regulation mechanism and pathway of gastric cancer progress, and they have been widely used in the field of gastric cancer research. In recent years, tumor genomics and proteomics have been widely used in biomedical and clinical research. Since the rise of gene chips and microarray technology, people have used these technologies to find new disease subclasses (17, 18), identify new tumor markers (19, 20), distinguish tumor grades (21), and predict the prognosis of the disease. For example, Wang et al. found that the increased expression of INHBA was related to the low survival rate of patients with gastric cancer through gene enrichment analysis (22). Liu et al. confirmed that extracellular matrix receptors and cell cycle signaling pathways may play an important role in gastric cancer (23). Wnt signaling pathway may lead to carcinogenesis by stimulating the migration and invasion of gastric cancer cells (24). β-Catenin is frequently mutated in gastric cancer (25). Fze3 is overexpressed in 75% of gastric cancer tissues and hsfrp is downregulated in 16% of gastric cancer tissues, indicating that the expression of fze3 and hsfrp in this pathological tissue is often changed (26). Highly recombinant Shh induces the migration and invasion of gastric cancer cells by regulating tissue growth factor (TGF), which plays a role in the alk5–smad3 pathway (27). LOXL2 can promote tumor invasion through the Src/FAK signaling pathway, and its expression in gastric cancer is significantly increased (28). The loss of embryonic liver cell lining protein (ELF) can destroy the TGF-mediated signal pathway by interfering with the localization of Smad3 and Smad4 and lead to gastric cancer (29). The increase of BMP-2 concentration can significantly improve the motility and invasiveness of gastric cancer cells (30). The upregulation of cycox-61 may lead to the progression of gastric cancer. Interleukin-6 induces the invasion of gastric cancer cell line AGS cells through the activation of the c-Src/RhoA/ROCK signaling pathway (31).

Although the cost of large-scale sequencing data is decreasing and the speed is increasing, the number of clear gastric cancer-related genes remains small. A large number of multi omics data of gastric cancer have been accumulated. It is an important means to fully understand the genetic mechanism of gastric cancer to preliminarily screen potential genes through large-scale data mining algorithms and then verify them one by one through biological experiments. Systems biology aims to study the interaction of various molecules with different structures and functions at the overall level of organisms, and then add computational methods to describe and predict biological functions (32, 33), phenotypes, and behaviors. Most of these methods are based on networks (34, 35). These computational methods have been widely used in the discovery of disease-related genes (33, 35–39), genetic mechanism (40, 41), gene expression (37, 40), protein function (42, 43), metabolic association (44, 45), and drug target (46, 47). Therefore, in this paper, we developed a novel method named “DBN-GTN” to identify gastric cancer-related genes in a large scale. This method is based on the thought of systems biology. It used multiple features of genes and gastric cancer to identify the patterns of gastric cancer-related genes, which can be used to find more gastric cancer-related genes.

## Method

### Workflow

**Figure 1:**
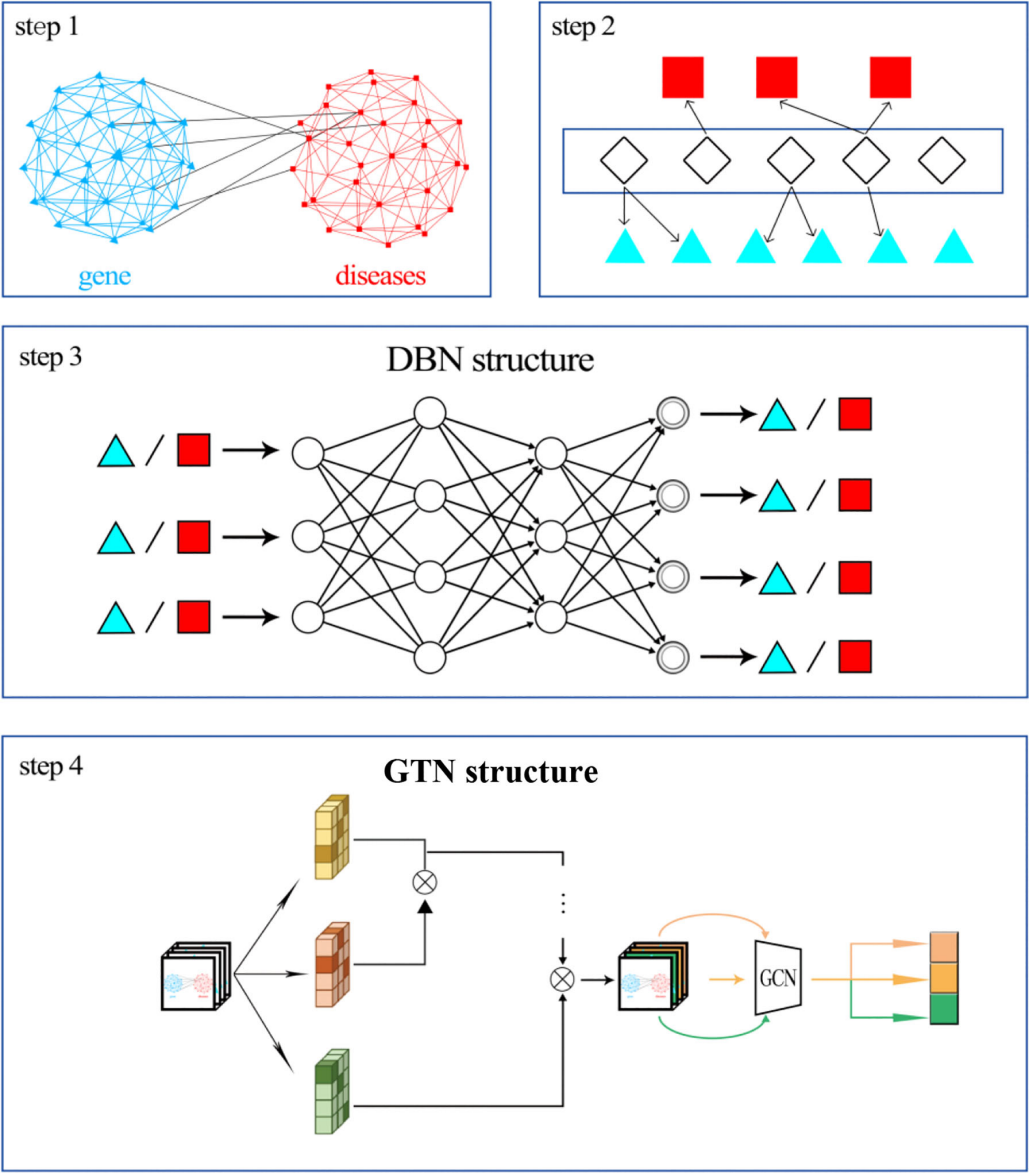
Workflow.

We firstly constructed a disease similarity network and a gene interaction network. We connected the two networks together based on the known relationship between diseases and genes. For example, the public databases have shown that the EGFR gene has a relationship with gastric cancer. Then, the node “gastric cancer” can be connected to the node “EGFR”. Finally, we can obtain a heterogeneous network. Then, we should extract the features of diseases and genes, respectively. We used the relationship between miRNAs and both diseases and genes as the features. Therefore, gene feature is the regulatory relationship between the gene and all miRNAs. Disease feature is the known relationship between the disease and all miRNAs. Then, the deep belief network (DBN) was applied to reduce the dimension of features. Finally, the graph transformer network was implemented to train the model and predict gastric cancer-related genes. The workflow is shown in **Figure 1**.

### Construction of Heterogeneous Network

Firstly, we need to calculate the similarity of diseases. Disease ontology (DO) was applied to explore the relationship between different diseases. Every disease term in DO is related to some molecular components (such as genes, proteins, small molecules, and drugs), which are usually called annotation entities of diseases. The similarity between the two diseases is also related to their common ancestors. The similarity of two diseases from the same ancestor node is usually greater than that of two diseases that do not belong to the same ancestor node. Therefore, the similarity of two diseases can be calculated by calculating the amount of information of two disease ancestor nodes. Similarly, in DO, each disease is related to its annotation entity. The similarity between the two diseases can also be calculated by calculating the relationship between their annotation entities.

Then, we need to obtain gene interaction information. We downloaded gene interaction information from HumannetV2.0 (48). The genes that can interact with each other can be connected in the gene network.

Finally, we need to connect these two networks based on the known relationship between diseases and genes. The DisGeNet database (49) was used to obtain the associations between diseases and genes. Based on the information reported by DisGeNet, we can build a heterogeneous network of diseases and genes.

### Feature of Diseases and Genes

DIANA-TarBase v8 (50) collected decade-long experimentally supported miRNA–gene interactions. Using this database, we obtained the relationship between genes known to be related to disease and miRNAs. Each miRNA is one dimension of a gene feature. If a gene is reported to be regulated by the miRNA, the feature value of this gene in this characteristic dimension is 1.

Mir2disease (51) contains 349 miRNAs, 163 diseases, 3,273 miRNAs, and the association information between diseases. Using this database, we obtained the relationship between miRNAs and diseases similar to gastric cancer. Each miRNA is one dimension of a disease feature. If a disease is reported to be related to the miRNA, the feature value of this disease in this characteristic dimension is 1.

### Dimensionality Reduction by Deep Belief Network

In order to reduce the feature dimension of miRNA, we constructed a DBN network architecture based on Restricted Boltzmann Machine (RBM) for miRNA feature encoding. Each RBM is a layer in the DBN network architecture, and the DBN-based miRNA feature encoding method contains a total of 3 layers of RBMs.

First, the variables in RBM are divided into hidden variables and observable variables. Among them, the observable variables are the features of miRNAs. The observable and hidden variables are represented by the observable layer and the hidden layer, respectively. The nodes in the RBM layer are not connected, and all the nodes in the adjacent RBM layers are connected to each other. This connection method is consistent with the fully connected neural network.

Unsupervised learning is difficult because the distribution of input miRNA features is unknown. Based on the conclusions of statistical mechanics, we describe the probability distribution with an energy-based model. An RBM is composed of miRNA features and latent variables, whose energy function is defined as:


(1) $$E(v,h)=-\sum_{i}a_{i}v_{i}-\sum_{j}b_{j}h_{j}-\sum_{i}\sum_{j}v_{i}w_{ij}h_{j}=-a^{T}v-b^{T}v-v^{T}Wh$$

Where the feature of genes can be represented *v* = [*v*_1_, *v*_2_,…*v_m_*]*^T^* ; *h* is the random vector *h* = [*h*_1_, *h*_2_, …*h_n_*]*^T^ W* is the matrix of weight. Both *a* and *b* are bias.

With the energy function, the joint probability between the original feature of a gene and the feature after dimensionality reduction can be defined, and the conversion from the visualized node to the hidden node can be realized. Denote the joint probability distribution as *p*(*v*, *h*), which is calculated as follows:


(2) $$p(v,h)=\frac{1}{Z}\exp(-E(v,h))=\frac{1}{Z}\exp(a^{T}v)\exp(b^{T}h)\exp(v^{T}Wh)$$

Where $Z=\sum_{v,h}\exp(-E(v,h))$

is the partition function and can also be called normalization coefficient.

### Prediction of Gastric Cancer-Related Genes by the Graph Transformer Network

Since our network is a heterogeneous network of diseases and genes, there are multiple types of meta-paths in it. The first step is to select edge types from the adjacency matrix *A*. Then, we need to do matrix multiplication of two selected adjacency matrices to learn a novel meta-path network *A* ^(1)^. This new adjacency matrix can be calculated as the sum of candidate adjacency matrices based on weight. The addition process is based on 1*1 convolution with the activation function softmax.


(3) Q=F(A,W)=σ(A,    softmax(W))

*σ*() represents a convolutional layer and *W* is the weight of it.

In this way, GTN can generate new meta-path adjacency matrices (52). Then, we can implement graph convolutional network (GCN) on these adjacency matrices. Each GCN layer can be calculated as:


(4) $$H^{\left(l+1\right)}=\sigma (D^{-\frac{1}{2}}AD^{-\frac{1}{2}}H^{l}W^{l})$$

Finally, each node in GTN can be encoded as:


(5) $$Z=∥\sigma (D^{-1}AXW)$$

## Results

### Compare With Traditional Methods

We obtained a total of 435 genes that are reported to be related to gastric cancer. We randomly selected 435 genes as the positive samples and selected part of the remaining genes as negative samples to train the model. We compared DBN-GTN with several traditional methods, which include support vector machine (SVM), back-propagation artificial neural networks (BP-ANN), naive Bayes, and random forest. Since these methods do not have the ability to encode a network, we simply combined the features of genes and diseases to construct a disease–gene pair. We input these disease–gene pairs into these traditional methods and build models to predict gastric cancer-related genes. The performance of these methods is shown in **Table 1**.

**Table 1:** AUC and AUPR of traditional methods and DBN-GTN.

Method	AUC	AUPR
DBN-GTN	0.93	0.86
SVM	0.78	0.68
BP-ANN	0.80	0.73
Naive Bayes	0.72	0.63
Random Forest	0.75	0.69

As we can see in **Table 1**, DBN-GTN performed best among these methods. The main reason why the accuracy of our analysis of DBN-GTN is significantly higher than other methods is that it considers the association between diseases and the interaction between genes, while other traditional methods are limited by their own shortcomings and cannot incorporate this information into the models.

### Compare With Similar Methods

**Figure 2:**
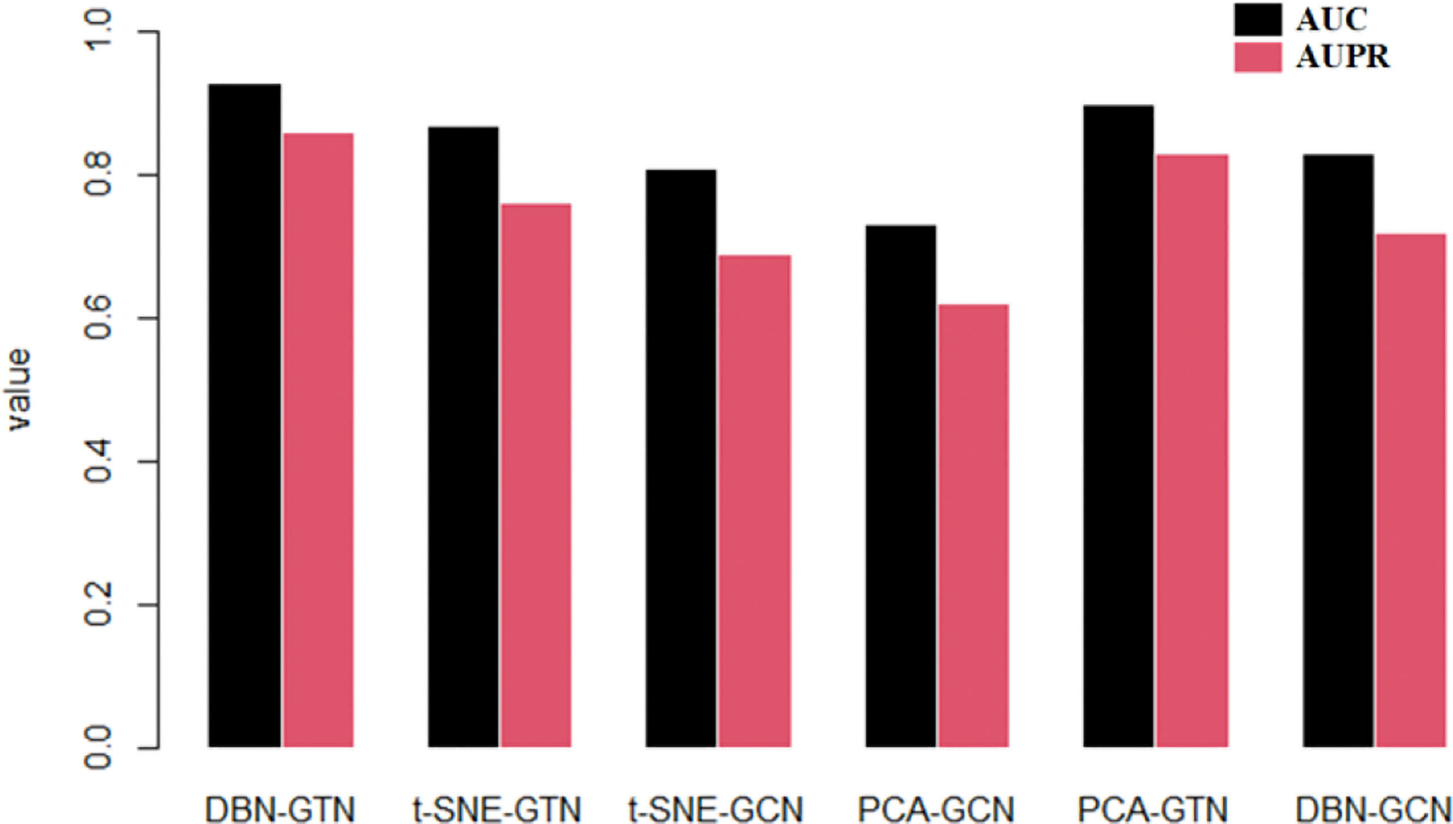
AUC and AUPR of DBN-GTN and similar methods.

Two methods make up the DBN-GTN, and we try to replace the two methods with similar methods to test whether the accuracy of the method is the highest. DBN mainly plays the function of dimensionality reduction, and principal component analysis (PCA) and t-distributed stochastic neighbor embedding (t-SNE) have a similar function. Therefore, we try to use these two methods to replace DBN and test the performance. In addition, GCN can be used to encode a homogeneous network. To compare the difference between encoding a heterogeneous network and encoding two homogeneous networks separately, we used GCN to replace GTN. GCN was implemented to encode a gene interaction network and a disease similarity network, respectively. Then, the features of genes and diseases are combined together to train the GCN model. The experimental results are shown in **Figure 2**.

As we can see from **Figure 2**, DBN-GTN performed best among these methods and t-SNE-GCN performed worst. From the impact of dimensionality reduction on accuracy, DBN outperforms t-SNE and PCA, and t-SNE has the worst accuracy. This is because PCA can manually select the amount of information contained after dimensionality reduction, while t-SNE can only reduce the data to 2 to 3 dimensions. From the perspective of the influence of the coding network method on accuracy, the performance of GTN is better than that of GCN. This is because GTN can encode heterogeneous networks and obtain more information than two homogeneous networks by GCN.

## Conclusion

Biologists discovered some genes related to gastric cancer through large-scale transcriptome and genome sequencing. These results suffer from sample heterogeneity and insufficient sample size. At the same time, these experiments also cost a lot of time and money. Therefore, from the perspective of systems biology, this paper mines the association patterns between diseases and genes, and establishes a model through deep learning algorithms to identify large-scale gastric cancer-related genes. Although a large number of previous studies have used computational methods to identify gastric cancer-related genes, most of these methods focus on extracting information from homogeneous networks and cannot fully incorporate the association between diseases and genes into the model. In this paper, we established a disease similarity network and a gene interaction network, and connected the two networks through the known correlation between the two to form a disease–gene heterogeneous network. At the same time, we extracted the features of the disease and gene based on their relationship with miRNAs. In other words, a bridge between diseases and genes is established through miRNAs. We employ deep belief networks for feature dimensionality reduction and GTN for heterogeneous network encoding. We call this method DBN-GTN. We compare the accuracy of this method with four traditional methods and five similar methods. Experimental results show that DBN-GTN outperforms our chosen traditional method and similar methods, which shows that DBN-GTN is superior in the task of large-scale identification of gastric cancer genes. This paper provides support to further explain the genetic risk, susceptibility, and drug screening of gastric cancer.

## Data Availability Statement

The datasets presented in this study can be found in online repositories. The names of the repository/repositories and accession number(s) can be found in the article/**Supplementary Material**.

## Ethics Statement

Ethical review and approval was not required for the study on human participants in accordance with the local legislation and institutional requirements. Written informed consent for participation was not required for this study in accordance with the national legislation and the institutional requirements.

## Author Contributions

Experimental design: YC, XS, and JY. Data analysis: YC and XS. All authors contributed to the article and approved the submitted version.

## Conflict of Interest

The authors declare that the research was conducted in the absence of any commercial or financial relationships that could be construed as a potential conflict of interest.

## Publisher’s Note

All claims expressed in this article are solely those of the authors and do not necessarily represent those of their affiliated organizations, or those of the publisher, the editors and the reviewers. Any product that may be evaluated in this article, or claim that may be made by its manufacturer, is not guaranteed or endorsed by the publisher.
